# Application of the MAHDS Method for Multiple Alignment of Highly Diverged Amino Acid Sequences

**DOI:** 10.3390/ijms23073764

**Published:** 2022-03-29

**Authors:** Dimitrii O. Kostenko, Eugene V. Korotkov

**Affiliations:** Research Center of Biotechnology of the Russian Academy of Sciences, Institute of Bioengineering, Bld. 2, 33 Leninsky Ave., 119071 Moscow, Russia; info@fbras.ru

**Keywords:** multiple alignment, dynamic programming, amino acid sequence

## Abstract

The aim of this work was to compare the multiple alignment methods MAHDS, T-Coffee, MUSCLE, Clustal Omega, Kalign, MAFFT, and PRANK in their ability to align highly divergent amino acid sequences. To accomplish this, we created test amino acid sequences with an average number of substitutions per amino acid (*x*) from 0.6 to 5.6, a total of 81 sets. Comparison of the performance of sequence alignments constructed by MAHDS and previously developed algorithms using the *CS* and *Z* score criteria and the benchmark alignment database (BAliBASE) indicated that, although the quality of the alignments built with MAHDS was somewhat lower than that of the other algorithms, it was compensated by greater statistical significance. MAHDS could construct statistically significant alignments of artificial sequences with *x* ≤ 4.8, whereas the other algorithms (T-Coffee, MUSCLE, Clustal Omega, Kalign, MAFFT, and PRANK) could not perform that at *x* > 2.4. The application of MAHDS to align 21 families of highly diverged proteins (identity < 20%) from Pfam and HOMSTRAD databases showed that it could calculate statistically significant alignments in cases when the other methods failed. Thus, MAHDS could be used to construct statistically significant multiple alignments of highly divergent protein sequences, which accumulated multiple mutations during evolution.

## 1. Introduction

Algorithms for multiple sequence alignment (MSA) are widely used in modern molecular biology for numerous purposes, including evolutionary studies and molecular modeling. Thus, the publication describing the ClustalW multiple alignment algorithm [1] is among the top 10 most cited publications [2]. MSA is indispensable when it is necessary to determine the relatedness of polypeptide sequences containing substitutions and/or insertions/deletions (indels) of amino acid residues, and MSA algorithms are fundamental tools for the construction of phylogenetic trees, calculation of profiles for hidden Markov models (HMMs), and search for common motifs. An important application of MSA methods is the prediction of secondary and tertiary structures of polypeptides and RNA, as well as their molecular functions and interactions [3,4].

In general, the construction of an MSA is an NP-complete problem, for which no direct solution by dynamic programming methods is possible in a reasonable time, even using modern supercomputers [5]. Therefore, many heuristic methods for performing MSA in a relatively short computational time have been developed. Among them, one of the most popular is the progressive procedure based on the construction of a binary (guiding) tree in which sequences are presented as leaves [6]. Then, MSA can be built through a series of pairwise alignments added according to the branching order in the path tree. Methods that employ the progressive approach include ClustalW [1] and its variant Clustal Omega, based on HMM for protein alignments [7], MAFFT [8], and T-Coffee [9]; the latter uses a paired alignment library, which allows for the construction of more accurate multiple alignments than those produced by ClustalW or Clustal Omega.

Iterative methods such as PRRN/PRRP [10] and MUSCLE [11] have been developed with the aim to reduce errors in constructing progressive alignments, which is achieved by repeatedly rebuilding the generated MSAs. Iterative methods revert to pairwise alignments, which can optimize the objective function and improve the quality of MSA.

Some MSA algorithms such as SAM [12] and HMMER [13] use HMM. Compared to progressive methods, HMM-based methods often calculate more significant alignments, which is achieved by rebuilding the alignment each time a new sequence is added.

There are also other methods for calculating MSA, such as genetic algorithms [14], annealing modeling [15], and phylogeny-aware gap placement [16,17]. Recently, we have developed an algorithm for multiple alignments of highly diverged sequences (MAHDS), which is based on the optimization of random position weight matrix (PWM) images of MSAs [18,19,20]. To optimize random patterns in MSA calculation, the MAHDS method uses a genetic algorithm and two-dimensional dynamic programming rather than pairwise equalization; as a result, multiple alignments for highly divergent nucleotide sequences can be obtained. The application of MAHDS to random nucleotide sequences shows that it can calculate statistically significant multiple alignments of sequences, for which the number of random base substitutions per nucleotide (*x*) ranges from 0 to 4.4. At the same time, the other methods such as ClustalW [21], Clustal Omega [7], MAFFT [22], T-Coffee [9], Muscle [11], and Kalign [23] could do it for x from 0 to 2.4 [18], indicating that for highly divergent nucleotide sequences (*x* = 2.4–4.4), the MAHDS algorithm is the only method capable of building statistically significant alignments. Previously, MAHDS has been used to construct MSAs of highly diverged promoter sequences (*x* = 3.6) from different genomes [19,24,25], which made it possible to identify a large number of potential promoters in rice genes [25]. 

In this study, we used the MAHDS method for the calculation of MSAs of protein sequences. The application of MAHDS to the alignment of random amino acid sequences with different *x* showed that the method could produce statistically significant MSAs for sequences with *x* ranging from 0 to 3.6. The alignments constructed by MAHDS and the other methods mentioned above were performed using the column score (*CS*) test [26,27,28], *Z*-score, and the benchmark alignment database (BAliBASE) (reference set 10) containing 218 protein families. Although, according to the *CS* criterion, the quality of the alignments built by the MAHDS method was somewhat lower than that of the other algorithms, it was compensated by greater statistical significance. Furthermore, in the alignment of 21 highly diverged protein families from the Pfam and HOMSTRAD databases, MAHDS could calculate statistically significant MSAs of more families than the other methods. Our results demonstrate that the developed MAHDS algorithm can be applied to analyze the phylogenetic relationships of evolutionary distant proteins.

## 2. Results

The performance of the MAHDS algorithm was compared with that of the MSA methods provided by EMBL-EBI resources, including T-Coffee, MUSCLE, PRANK, Clustal Omega, Kalign, and MAFFT, for which the web application programming interface is available. The following test data were used for analysis: benchmark alignment database (BAliBASE) [27,28,29], artificial sequences with certain properties, and protein families with low average sequence identity in full alignment.

The quality of alignments was evaluated using the *CS* [26,27,28] and *Z*-score criteria. In the calculation of the *CS*, the columns of the alignment are considered. If all remainders in the *i*-th column are aligned as in the reference alignment, then *C_i_* = 1; otherwise, *C_i_* = 0. The formula for calculating the *CS* is:

(1) $$CS=\frac{\sum_{i=1}^{L}C_{i}}{L}$$

where *L* is the number of columns in the evaluated alignment. As in order to use the *CS*, it is necessary to have reference alignments, the *CS* was applied only to evaluate the BAliBASE using its tools bali score and bali_score_reliable; the former considers all sequences, whereas the latter ignores sequences with discrepancies (annotated with SEQERR features [30]). The MAHDS algorithm uses the following parameters: gap opening penalty (*d*), gap extension penalty (*e*), *R_L_*, and *K_d_*. *R_L_* is the multiplier parameter, which can be used to scale *R^2^* (Formula (2)), and *K_d_* is the equivalent of an expected *E* score value features [31], which defines the accuracy of determining the start and end of the local alignment (Formula (3)). Therefore, the primary task in testing MAHDS was to find a biologically relevant combination of *d*, *e*, *R_L_*, and *K_d_*.

### 2.1. Testing MAHDS Performance Using the BAliBASE

We used BAliBASE reference set 10, comprising 218 protein families. However, because of restraints set on the file size and the number of sequences by EMBL-EBI services, it was necessary to exclude five families (BBA0039.fasta, BBA0101.fasta, BBA0134.fasta, BBA0190.fasta, and BBA0213.fasta) for which alignment could not be performed by at least one of the compared methods. As a result, we considered the remaining 213 protein families and calculated the average *CS* for the MSAs as an indicator of the biological correctness, which should be maximized when choosing the parameters. At the same time, we tried to ensure that Z (Methods, Formula (7)) was close to the maximum.

We carried out a series of experiments for different values of parameters *d*, *e*, and *K_d_* at fixed *R_L_* = 5.0, which can be chosen arbitrarily, as *d*, *e*, and *K_d_* would then be adjusted accordingly. Initially, we considered *e* = 0.25 *d* as the optimal ratio, which was previously established for the application of MAHDS to DNA sequences [18], and estimated *Z* and *CS* scores for the sets of *d* values {8, 10, 12, 16, 20, 24, 28, 32, 36, 40, 44, 48, 52, 56} and *K_d_* values {−0.1, −1, −2}. However, it appeared that with *e* = 0.25 *d* there was a discrepancy between the peaks in the *CS* and *Z*; therefore, an additional series of experiments was conducted with varying *e*-to-*d* ratios (Table 1).

The results indicated that the combination *d* = 40, *e* = 1, and *K_d_* = −1.0 was the optimal for protein sequence alignment and *Z* estimation with MAHDS. Then, we aligned the 213 protein families by the other methods and estimated the average *Z* (calculated as described in Section 4.6.1) and *CS* (Table 2). The results showed that the MAHDS algorithm was superior to the compared methods in terms of statistical significance (*Z*) but inferior in terms of *CS*.

### 2.2. Determining the Significance Threshold for Z

As a rule of thumb, paired alignment is considered statistically significant at *Z* ≥ 8 [32]. For MAHDS, the threshold value was 6 [18] or 10 [33], depending on the nature of the sequences; however, it has been determined for the alphabet of four nucleotides and may not be relevant for protein sequences composed of 20 amino acids.

To determine the threshold of statistical significance *Z_t_*, we created random sequences. To ensure that the number of aligned sequences and their average length did not affect *Z*, two datasets were generated: *H*_1_ containing 100 subsets of 100 random sequences, each 600 amino acids long, and *H*_2_ containing 100 subsets, including 20, 40, and 40 random sequences of 600, 900, and 300 amino acids, respectively. For each *H*_1_ and *H*_2_ subset, we built sequence alignments using the MAHDS algorithm and calculated *Z*. For set *H*_1_, $\bar{Z_{H_{1}}}$ = 4.147 and $\sqrt{D_{H_{1}}}=$ 2.11, whereas for set *H*_2_, $\bar{Z_{H_{2}}}$ = 4.04 and $\sqrt{D_{H_{2}}}=$ 1.78. These results showed that the variations in sequence lengths did not affect *Z*, indicating that the same *Z_t_* could be used for sequences of different lengths. Therefore, we chose *Z_t_* = 10.0, which was approximately equal to the average value of $\bar{Z_{H_{1}}}+3\sqrt{D_{H_{1}}}$.

### 2.3. Testing MAHDS Performance Using Artificial Sequences

To compare methods for constructing multiple alignments, we created 81 sets, each containing 100 artificial sequences *Des*(*i*) generated from ancestor sequence *Anc* of length (*L*) = 600 symbols (Methods, Section 4.7). To create *Des*(*i*), the following random changes were made to *Anc*: the numbers of insertions or deletions were 2, 5, or 10, the indel length was 1, 5, or 20, and the number of substitutions per amino acid was 0.3, 0.6, 0.9, 1.2, 1.5, 1.8, 2.1, 2.4, or 2.7. In accordance with Formula (11), the average number of substitutions per symbol between sequences in sets *Des* was 0.6, 1.2, 1.8, 2.4, 3.0, 3.6, 4.2, 4.8, or 5.4.

We aligned each of the 81 artificial *Des* sequence sets using MAHDS, T-Coffee, MUSCLE, Clustal Omega, Kalign, MAFFT, and PRANK and calculated *Z* values (Table 3, Table 4, Table 5 and Table 6; only *x* values for which Z > 0.0 are shown).

The results indicated that MAHDS could produce statistically significant alignments even if the evolutionary divergence between the aligned sequences (*x*) was 4.8 substitutions per amino acid; however, it applied to sequences with relatively small numbers of short indels. In other cases, MAHDS can build multiple alignments for *x* ≤ 3.6 (Table 3). At the same time, most of the other tested methods could not produce statistically significant results for sequences with *x* > 1.2 (Table 4, Table 5 and Table 6). MUSCLE showed the best performance among the other tested methods, being able to build statistically significant alignments for sequences with *x* ≤ 2.4 and small numbers of short indels; however, with five indels of 20 residues each, the *x* value dropped to ≤ 1.2 (Table 5).

### 2.4. Testing MAHDS Performance on Protein Families with Low Identity

As shown above, MAHDS could construct statistically significant MSAs for artificial sequences with *x* ≤ 4.8, whereas the other tested algorithms could not produce statistically significant alignments at *x* > 2.4. In the next experiment, we verified the efficiency of MAHDS in aligning divergent sequences with a high *x* value using protein families from Pfam and HOMSTRAD databases with a low percentage of identity.

In total, 21 families with an identity of less than 20% were aligned using the MAHDS algorithm, and the constructed MSAs were compared with those built using T-Coffee and MUSCLE chosen because they could produce statistically significant alignments for artificial sequences with the largest *x* among the other tested methods. These 21 families are shown in Table 7, and the names of protein families received from Pfam are shown in Table 8.

The performance of MAHDS, T-Coffee, and MUSCLE was evaluated based on statistical significance (*Z*), the number of gap openings, and the total number of gaps in alignments. The results shown in Table 7 indicated that, for most analyzed families, MAHDS could build alignments of higher statistical significance than the other two methods, with the exception of PF02950, PF09624, PF09987, and PF19443 families, for which the MSAs constructed by MUSCLE had greater *Z* (however, it should be noted that for PF02950, both MAHDS and MUSCLE showed *Z* < 10.0). At the same time, only MAHDS was able to construct statistically significant alignments for PF00915, PF10846, PF10895, and PF13944 families. Furthermore, there was a tendency for MAHDS to use fewer gap openings, although the series of gaps tended to be longer than for the other two methods.

## 3. Discussion

Here, we described the application of our MAHDS method, which was previously used for the alignment of DNA sequences [18,19,20], to the construction of statistically significant alignments of highly divergent protein sequences that have accumulated multiple amino acid substitutions and/or indels. Comparison with the currently available MSA algorithms revealed that MAHDS could produce significant alignments of model sequences with low identity (*x* up to 4.8), whereas the other methods (T-Coffee, MUSCLE, Clustal Omega, Kalign, MAFFT, and PRANK) could only carry this out for less diverged sequences (*x* < 2.4). The superior performance of the MAHDS method is due to the use of the progressive algorithm instead of pairwise alignment and calculation of MSA based on patterns of random alignments. In this case, it is possible to replace N-dimensional dynamic programming, which requires significant computational resources, with two-dimensional dynamic programming. Such an approach enabled the construction of more statistically significant MSAs for 21 protein families with a low percentage of identity than could be performed by the other methods.

We made an assessment of the speed of the MAHDS. For 100 amino acid sequences with a length of 100, 400, and 700 amino acids, the running time of the algorithm was 58, 851, and 2370 s, respectively. This suggests that the calculation time grows approximately in a quadratic dependence on the length of the sequences. For sets of amino acid sequences containing 100, 400, and 700 sequences having a length of 100 amino acids, the multiple alignment building times were 58, 333, and 629 s. These results show that there is an approximately linear relationship between the time required to build an alignment and the number of sequences. For calculations, two computing nodes were used. Each computing node includes two Intel Xeon Gold 6240 CPUs. Each processor has nine physical cores, i.e., we used 36 cores.

The developed algorithm made it possible to calculate the statistical significance of any alignment and compare the main methods used in terms of the statistical significance of the alignments that they can construct for protein families. Statistical significance in our work is estimated by the Monte Carlo method based on the generation of random multiple alignments. Using such a Z-score allows us to make direct probabilistic estimates based on which we can filter out statistically insignificant multiple alignments. Such probabilistic estimates are difficult to make using currently used object functions. Such functions include sum of pairs, minimum entropy, or NorMD [34,35].

A question arises regarding when the developed MAHDS method for MSA of amino acid sequences should be applied and what results could be expected. Obviously, MAHDS would be most useful for analysis of those amino acid sequences that have accumulated multiple mutations (*x* > 2.4), thus facilitating the prediction of the biological role for the amino acid sequences of unknown function [36]. For this, MSA of the protein family in question could be calculated using MAHDS and then analyzed by similarity search using the profile method [37] or HMM [38]. However, the quality of the MSA can strongly influence the search for statistically significant similarities [39]. Therefore, it is important to use MAHDS to compare highly divergent sequences and calculate MSA. Probably, it could be possible to identify residues, for which the biological function is either unknown or the reliability of the annotation is questionable.

The MAHDS method could also be used to reconstruct the evolutionary history of sequences. In this respect, MAHDS can provide an advantage by constructing statistically significant alignments and tracing similarity between evolutionary distant sequences with *x* = 2.4–4.8, thus resolving the problem of MSA uncertainty [40] and revealing previously unknown evolutionary relationships between different protein families.

By disclosing similarities among highly diverged proteins, MAHDS can also be applied to predict various parameters of protein conformation, including fold recognition and structure-based functional activity [41,42,43], which can help in the construction of three-dimensional models for various proteins [44].

We have developed a site, http://victoria.biengi.ac.ru/mahds/main (3 June 2020), where the user can build multiple alignments for any set of amino acid sequences using MAHDS. Our work was performed using resources of the NRNU MEPhI high-performance computing center.

## 4. Materials and Methods

### 4.1. Algorithm to Calculate Multiple Alignment

The MAHDS method was developed early for the multi alignment of promoter sequences and DNA sequences with weak similarity [18,19,20]. This method uses mathematical approaches and programs that were previously created to search for tandem repeats in various sequences [33,45,46,47,48,49]. In the present work, the site http://victoria.biengi.ac.ru/mahds/main (3 June 2020) was used to construct a multiple alignment by the MAHDS method for various amino acid sequences.

Let us briefly consider the mathematical algorithm that underlies the MAHDS method. This algorithm is described in detail in [18]. Let us calculate multiple alignment for a set of *N* sequences denoted as *SI*. The optimal multiple alignment (*MA*) for this set is characterized by a maximum of some function *Ψ* (*maxΨ*), which is calculated from *MA*. Then, we define the image of multiple alignment (*IMA*) as a function of *MA*: *IMA* = *f(MA*) and also consider an inverse function to *f*: *f*^−1^(*IMA*) = *MA*, which allows us to construct *MA* from *IMA* and the *SI* sequences. Specifying functions *f* and *f*^−1^ makes it possible to unambiguously convert *MA* to its image (*IMA*) and vice versa, i.e., to establish one-to-one correspondence between *MA* and *IMA*.

Several existing algorithms, including progressive alignment [6], HMM [7], and others [3,4] can produce alignment *MA*’, which is close to the optimal *MA*. In these methods, the construction of multiple alignment is defined by function *ζ*: *MA’* = *ζ*(*SI*). The main disadvantage of such an approach is that the alignment is based on pairwise comparisons of sequences from set *SI*, which precludes building of statistically significant *MA*’ when the number of nucleotide substitutions *x* is greater than 2.4 [18]. A statistically significant *MA*’ has a probability *P*(*Ψ > Ψ*_0_) < α, where *Ψ*_0_ is the value of function *Ψ* for *MA*’. This probability is calculated by aligning a large number of sets *SR*, each of which comprising a series of randomly shuffled sequences of set *SI* and determining the distribution of function *Ψ* for this alignment. The value of α can be chosen as 0.05, if the alignment is built once.

However, there is another way of constructing *MA*’, which is not based on pairwise alignments and direct calculation of *MA’* = *ζ*(*SI*) but uses patterns (images) of random multiple alignments (*IMAR*) [18]. These images can be subjected to optimization using genetic algorithms in order to create from a random image *IMAR* such an image *IMA*_m_ that would be the closest to *IMA* = *f(MA).* The degree of closeness between *IMA*_m_ and *IMA* cannot be assessed, since *MA* and its image *IMA* = *f(MA*) are unknown at *x* > 2.4 for most protein families. However, we can arrive as close as possible to *IMA* by increasing the similarity measure between each sequence from set *SI* and *IMA*_m._, which can be carried out using global two-dimensional alignment. Each such comparison would produce a maximum value of similarity function *Fmax*(*i*) (*i* = 1, 2, ..., *N*), and the sum $mF=\sum_{i=1}^{N}Fmax(i)$ would be used as the measure of similarity between *IMA_m_* and *IMA*. The greater *mF* is obtained with the same global alignment parameters, the more similarity there is between *IMA_m_* and each sequence from set *SI* and the closer *IMA_m_* is to *IMA*. We will also use *mF* as an objective function when running a genetic algorithm to calculate *IMA_m_*, which then would be used to define multiple alignment: *MA_m_* = *f^−1^(IMA_m_*).

In this approach to calculate multiple alignment, we only need to define functions *f* and *f*^−1^. As function *f*, we can take the algorithm for creating a position weight matrix (PWM), which would be the image of multiple alignment, i.e., *IMA* = PWM and *IMA*_m_ = PWM_m_ (described below in Section 4.2). As function *f*^−1^, we take the algorithm for the global alignment of PWM_m_ and each sequence from set *SI* (described earlier [50] and in Section 4.3 and Section 4.4). As a result, it is possible to build multiple alignment with good accuracy using PWM_m_. Since the sequences in set *SI* can have different lengths, we calculate the average length $\bar{L}$ of the sequence in the set and create a PWM in the range from $0.9\bar{L}$ to $1.1\bar{L}$.

**Figure 1:**
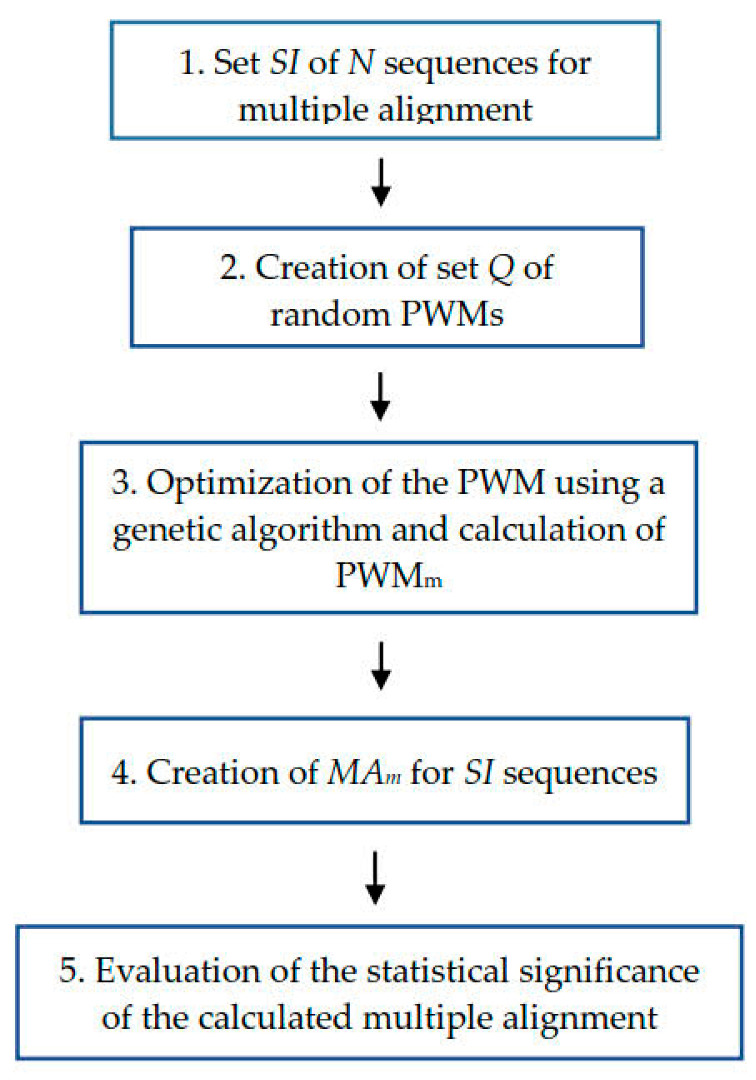
Diagram of the algorithm for multiple alignment of amino acid sequences shown in Section 4.1. Set of *N* sequences for multiple alignment is denoted as *SI*. *Q* is set of random PWMs (see Section 4.2). PWM_m_ is matrix that has the maximum value of the similarity function when aligning sequences from the set *SI* (see Section 4.3 and Formula (4)). *MA_m_* is a multiple alignment built for sequences from *SI* set using PWM_m_ (see Section 4.5 and Formula (5)).

Compared to the method described in [18], here we did not combine all sequences from set *SI* into one large sequence to calculate global alignment with the PWM but instead aligned the PWM with each *SI* sequence separately, which significantly improved the quality of MSA. Furthermore, the alphabet of *SI* sequences (*A_in_*) was expanded from 4 to 20 symbols to correspond to the number of amino acids so that protein sequences could be aligned. The scheme of the algorithm is shown in Figure 1.

At the beginning, we have *SI* sequences whose number is *N* (step 1). In step 2, we created a set (*Q*) of random PWMs, each of which served as a random image *IMAR*. In step 3, we used a genetic algorithm to optimize each PWM from set *Q* and determine PWM_m_ = *IMA_m_*. In step 4, we created multiple alignment *MA_m_* for sequences from set *SI*, which corresponded to the found PWM_m_. Finally, in step 5 we evaluated the statistical significance of the Monte Carlo alignment.

### 4.2. Creation of Set Q of Random PWMs

To create set *Q* of random PWMs, PWM rows were represented by 20 amino acids and columns by integers from 1 to *L*; thus, the PWM dimensions were 20 rows and L columns. A random PWM was obtained from a random amino acid sequence *S*_1_ of length *Lk* (*k* = 10^3^), in which the frequencies of amino acids corresponded with those in the sequences of set *SI*. Then, a sequence containing integers from 1 to *L* was generated, copied *k* times, and joined in tandem to yield sequence *S*_2_.

Next, we filled in frequency matrix *M*(*s*_1_(*i*), *s*_2_(*i*)) = *M*(*s*_1_(*i*), *s*_2_(*i*)) + 1 for all *i* from 1 to *L*x*k*, determined sums $x\left(i\right)=\sum_{j=1}^{L}M(i,j)$ and $y\left(j\right)=\sum_{i=1}^{20}M(i,j)$, and estimated probability $p\left(i,j\right)=x(i)y(j)/L^{2}$. After this, each PWM element was calculated as $pwm\left(i,j\right)=(M(i,j)-Lkp(i,j))/\sqrt{Lp(i.j)(1-p(i,j)}$. The procedure was repeated 500 times, yielding set *Q* (step 2, Figure 1).

Then, we performed transformation of the matrices from set *Q*, which was necessary to ensure that the distribution function of different *Fmax*(*i*) (*i* = 1, 2, ..., *N*) was similar in the alignment of sequences from set *SI* with each random PWM. For normalization purposes, two restrictions were imposed on PWMs:

(2) $$R^{2}=\overset{\left|A_{in}\right|}{\underset{i=1}{\sum }}\overset{L}{\underset{j=1}{\sum }}pwm{\left(i,j\right)}^{2}\;$$

(3) $$K_{d}=\overset{\left|A_{in}\right|}{\underset{i=1}{\sum }}\overset{L}{\underset{j=1}{\sum }}pwm(i,j)p_{1}(i)p_{2}(j)\;\;\;$$

where *p_1_(i)* and *p_2_(i)* are probabilities of amino acids in sequence *S*_1_ and of symbols in sequence *S*_2._, respectively. Upon transformation, any PWM used in the algorithm must be reduced to the given *R^2^* and *K_d_*. The matrix transformation procedure is described in detail in [33].

However, it should be noted that *R^2^* cannot be set constant, since in this case the number of cells in the PWM would increase and the cell values would not remain in approximately the same order (as needed) but would rather tend to approach zero in order to correspond to Equation (1). Therefore, *R^2^* was specified as a function of the period and cardinality of the *SI* sequence alphabet: *R^2^ = R_L_A_in_L*, where *R_L_* is the multiplier parameter, which can be used to scale *R^2^*.

As the sequences in set *SI* may have different lengths, we created sets of matrices *Q*(*L*) with length *L* ranging from $0.9\bar{L}$ to $1.1\bar{L}$ (see Section 4.1). Each *Q*(*L*) set contained 500 PWMs.

### 4.3. Using a Genetic Algorithm to Optimize PWMs

Then, we calculated PWM_m_ using a genetic algorithm described in detail in [18,33] (Figure 1, step 3). Briefly, PWMs of sets *Q*(*L*) with the size |Q| = 500 PWMs (Section 4.2) were considered as organisms. To calculate the objective function for each PWM from set *Q*(*L*), global alignment with each sequence from set *SI* was carried out. We calculated similarity function *Fmax(l)* at point (*L*1(*l*), *L*), where *l* is the sequence number in set *SI*, *L* is the number of columns in the PWM, and *L*1 is the length of the *l*-th sequence from set *SI*. Then, the *mF* value was calculated and used as an objective function for PWM matrices from set *Q*(*L*):

(4) $$mF(k)=\overset{N}{\underset{l=1}{\sum }}Fmax(l)\;$$

where *k* is the index of the matrix from set *Q*(*L*).

The PWMs were ranked according to *mF* in the descending order, and the matrix with the largest *mF* value, *mF*(1), was saved, whereas two PWMs with the smallest *mF* values were excluded from set *Q*(*L*). Then, we created two children from the remaining PWMs; as before [18,33], the offspring were generated by gluing of two parent matrices, which were chosen randomly. However, the probability of selecting a particular matrix increased with its *mF* value. In addition, random mutations were introduced into 50 randomly selected PWMs (except children created at this step) by replacing one random element by a random value evenly distributed in the interval from −10 to 10. After these changes, a new *Q*(*L*) set was generated, its matrices compared with the sequences from set *SI*, and a new vector *mF(k)* (*k* = 1, 2, ..., 500) was obtained. The procedure of modifying set *Q*(*L*) was iterated until *mF*(1) ceased to increase during the last 10 cycles. As a result of the genetic algorithm for set *Q(L*), *mF*(1) (denoted as *mF_L_*(1)) and the corresponding PWM were obtained.

The iterative procedure was performed in each *Q*(*L*) set for *L* from $0.9\bar{L}$ to $1.1\bar{L}$. Finally, we chose the *L* value for which *mF_L_*(1) was maximal; it was denoted as *L_m_* and the corresponding matrix as PWM_m_.

### 4.4. Global Alignment of PWMs from Set Q and Sequences from Set SI

Next, sequence *SI(l)* of length *L*1(*l*) was aligned with a PWM of number *k* from set *Q*(*L*). Sequence *SI(l)* was denoted as *S*3 and its elements as *s*3(*i*), where *i* = 1, 2, ..., *L*1(*l*). We also used sequence *S*4 with elements *s*4(*j*), where *j* = 1, 2, ..., *L*.

To construct the alignment of sequences *s*3(i) and *s*4(j), we used the global alignment algorithm [51] with an affine gap penalty function and PWM values from set *Q*(*L*) [33] instead of substitution matrices. Then, similarity function F was calculated as:

(5) $$F\left(i,\;j\right)=max\left\{\begin{array}{c} F(i-1,\;j-1)+PWM(s_{3}(i),\;s_{4}(j));\\ \left\{\begin{array}{c} F(i-1,\;j)-e,\;if\;F(i-1,\;j)\;was\;obtained\;from\;F(i-2,\;j)\\ F(i-1,\;j)-d,\;otherwise \end{array}\right.;\\ \left\{\begin{array}{c} F(i,\;j-1)-e,\;if\;F(i,\;j-1)\;was\;obtained\;from\;F(i,\;j-2)\\ F(i,\;j-1)-d,\;otherwise \end{array}\right.; \end{array}\right.$$

where *d* and *e* are gap opening and gap extension penalties, respectively. *Fmax(l)* = *F*(*L*1(*l*),*L*) (Section 4.2) was used as a measure of similarity between sequences *S*3 and *S*4. We also filled in matrix *F*’, where in each cell (*i*, *j*) we remembered the number of the cell from which we arrived at this cell using Formula (4). As a measure of similarity between set *SI(l)* and the PWM from set *Q*(*L*), we have chosen:

(6) $$mF(PWM)=\overset{N}{\underset{l=1}{\sum }}Fmax(l)\;$$

The PWM_m_ of length *L_m_* (Section 4.3) was aligned with set *SI(l)* using matrix *F*’, and the results were applied to calculate multiple alignments of sequences *SI(l)* (*l* = 1, 2, …, *N*), which were evaluated according to *mF(PWM_m_)* calculated with Formula (6).

### 4.5. Algorithm for Constructing Multiple Alignment

To obtain *MA_m_* for the *SI* sequences (Figure 1, step 4), we used PWM_m_ and a set of global alignments of the *SI(l)* sequences (*l* = 1, 2, ..., *N*,) with PWM_m_. For this, sequence *S*_4_ with elements *s*_4_(*j*) (*j* = 1, 2, ..., *L_m_*) was arranged horizontally, and under it the alignments of *SI(l)* sequences (*l* = 1, 2, ..., *N*) generated in Section 4.4 were written. If any of the *SI(l)* sequences had an insertion of *k* amino acids compared to sequence *S*_4_, then additional *k* columns were created for these amino acids in the *s*_4_(*j*) sequence (if they were not created for any other *SI(l)* previously). As a result, *MA_m_* was constructed.

### 4.6. Estimating the Statistical Significance of Multiple Alignments

#### 4.6.1. Assessing the Statistical Significance of MA_m_

To evaluate the statistical significance of the constructed multiple alignment *MA_m_* obtained with *PWM_m_* (Figure 1, step 5), we used the Monte Carlo method. First, we generated 300 sets of random sequences *Q_r_* through random shuffling of residues in the *SI* sequences. For each *Q_r_* we calculated *mF(PWM_m_)* using Formula (6), its arithmetic mean $\bar{mF(PWM_{m})}$ and dispersion *D*(*mF*(*PWM_m_*)), and, finally, *Z*:

(7) $$Z=\frac{mF(PWM_{m})-\bar{mF(PWM_{m})}}{\sqrt{D(mF(PWM_{m}))}}\;\;$$

A *Z* value greater than threshold *Z*0 indicated that the alignment obtained with *PWM_m_* was statistically significant.

#### 4.6.2. Estimating the Statistical Significance of an Arbitrary MA

The algorithm described above can be applied to evaluate the statistical significance of any alignment. Let us take an *MA* with length *K* containing α sequences, each denoted as $S_{{}_{5}}^{k}$ and its elements as $s_{{}_{5}}^{k}(j)$ (where *k* = 1, 2, ..., α and *j* = 1, 2, ..., *K*). Let us also introduce sequence *S*6 with elements *s*6(*j*), *j* = 1, 2, ..., *K* representing consecutive numbers from 1 to *K*; *S*6 would be used as column numbers for *MA*. First, we transform *MA* to remove those columns where the number of amino acids is less than α/2 and that of deletions is more than α/2; as a result, multiple alignment *MA*’ of length *K*’ is obtained. At the same time, sequence *S*6 is transformed by eliminating the numbers equal to those of the columns to be removed. As a result, we obtain sequence $S_{6}^{’}$ with length *K*’. Then, we calculate the amino acid frequency matrix for *MA*’ denoted as *V*(*i*,*j*), where *i* = 1, 2, …, 20 and *j* = 1, 2, …, *K*’; each element shows the number of type *i* residues at position *j* of *MA*’. Next, we calculate the PWM based on matrix *V*(*i*,*j*) as: $PWM(i,j)=(V(i,j)-p(i,j))/\sqrt{Up(i,j)(1-p(i,j)}$, where U is the total number of amino acids in MA’, $p(i,j)=X(i)Y(j)/U^{2}$, *X*(*i*) is the number of type *i* residues, and *Y*(*j*) is the total number of residues in the *j*-th column of *MA*’. Then, we transform *PWM*(*i*,*j*) according to Formulas (2) and (3).

After calculating *PWM′(i,j)*, the weight of *MA* can be determined. First, we compute the sum for all *k* = 1, 2, ..., α and *j* = 1, 2, ..., *K*: $Sum=\sum_{k=1}^{\alpha }\sum_{j=1}^{K}PWM’(s_{4}^{k}(j),s_{5}’(j))$. Next, we obtain the *Del*(*i*) vector, which shows the number of deletions with size *i* in *MA*. Finally, we calculate $F_{MA}=Sum-Del(1)d-\sum_{i=2}^{K}\left(Del(i)(d+(i-1)e\right)$, which is considered as the *MA* weight. Then, statistical significance should be evaluated by estimating mean $\bar{F_{MA}}$ and variance *D*(*F_MA_*). For this, symbols characterizing deletions are removed from $S_{{}_{5}}^{k}$ sequences, which are then randomly shuffled to yield $S_{{}_{Rand}}^{k}$ sequences, where *k* = 1, 2, ..., α, and the statistical significance is determined for matrix *PWM′(i,j)* and sequences $S_{{}_{Rand}}^{k}$ according to Formula (7), assuming that $SI=S_{{}_{Rand}}^{k}$, *N* = *K*, and *PWM_m_* = *PWM′(i,j)*. Thus, we calculate the *Z* value for *MA*, which enables comparison of different *MA*s by their statistical significance.

### 4.7. Creation of Artificial Sequences to Compare Different Methods of Constructing MAs

To compare the performance of MA methods T-Coffee, MUSCLE, Clustal Omega, Kalign, MAFFT, and PRANK with that of the MAHDS algorithm, we used artificial sequences, which can be created with a given number of amino acid substitutions and indels for convenience of analysis. To generate an artificial sequence, we first created a random ancestor sequence *Anc* of length *L* and then a set of descendant sequences *Des*(*i*) (*i* = 1, 2, …, 100) by adding a given number of random substitutions and/or indels to *Anc* in a random order. In case of random substitutions without indels, the lengths of child sequences were the same and equal to *L*, and the number of substitutions in each *Des*(*i*) sequence with respect to *Anc* was denoted as *s*_1_. Let us show that the presence of *s*_1_ random substitutions in each *Des*(*i*) sequence leads to 2*s*_1_ random substitutions between any two *Des* sequences.

When one random substitution is made in sequence *Anc*, the probability that in sequence *Des*(1) with the number of substitutions *s*_1_ a residue at position *j* will be changed is 1/*L*. Then, the probability that after *s*_1_ replacements residue *j* will not be replaced is:

(8) $$P_{0}={\left(1-\frac{1}{L}\right)}^{s_{1}}\;$$

We assume that amino acid substitutions during the creation of the *Des*(1) sequence occur with equal probability. For the rest of amino acids, it is likely that during the last replacement in a given position, the original amino acid will appear as substitution. The probability of falling into this category is:

(9) $$P_{1}=\frac{1}{\left|A_{in}\right|}\left(1-P_{0}\right)$$

where $\left|A_{in}\right|$ is 20. Thus, the probability that residue *j* in sequence *Des*(1) will match the residue at *Anc* is:

(10) $$P_{m}=P_{0}+P_{1}$$

The events described in Formulas (9) and (10) are applicable to all situations, when a residue at position *j* remains unchanged after random substitutions.

Now, let us consider the case when sequences *Des*(2) and *Des*(3) are independently generated from sequence *Anc* by making *s*_2_ random substitutions. Then, for both sequences, Formulas (8)–(10) can be presented as: $P_{02}={\left(1-\frac{1}{L}\right)}^{s_{2}}$, $P_{12}=\frac{1}{\left|A_{in}\right|}\left(1-P_{02}\right)$, $P_{m2}=P_{02}+P_{12}$. Let us take *P_m_*_3_ as a probability that in *Des*(2) and *Des*(3) two residues could match. If *P_m_*_3_ = *P_m_*, then the evolutionary distance between *Anc* and *Des*(1) is equal to that between *Des*(2) and *Des*(3), which means that, on average, the same number of amino acids coincide between *Anc* and *Des*(1) and between *Des*(2) and *Des*(3). In *Des*(2) and *Des*(3), the proportion of residues unchanged compared to *Anc* is *P_02_*; then, the proportion of residues preserved at the same positions of child sequences is $P_{03}=P_{02}^{2}$, since it is the probability for a residue to remain in both *Des*(2) and *Des*(3). Other amino acids matching in *Des*(2) and *Des*(3) include those that have been substituted with the same residues in both sequences, and the probability of such events is $P_{m4}=\sum_{k=1}^{20}{\left(1-P_{02}\right)}^{2}/{\left|A_{in}\right|}^{2}={\left(1-P_{02}\right)}^{2}/20$.

Finally, there is a probability that a residue that remained from *Anc* in *Des*(2) could match the one randomly replaced in *Des*(3) and vice versa. The sum of the two probabilities is: $P_{m5}=2\sum_{k=1}^{20}P_{02}(1-P_{02})/{\left|A_{in}\right|}^{2}=2P_{02}(1-P_{02})/20$. Then, $P_{m4}+P_{m5}$ = $\frac{1}{20}\left(1-P_{02}^{2}\right)$ and $P_{m3}=P_{02}^{2}+(1-P_{02}^{2})/20$. If *P_m_*_3_ = *P_m_*, then $P_{02}^{2}+(1-P_{02}^{2})/20=P_{0}+(1-P_{0})/20$, i.e., $P_{02}^{2}=P_{0}$. If the values for *P_02_* and *P_0_* are substituted, then:

(11) $$2s_{2}=s_{1}$$

Formula (11) means that the number of *s_2_* random substitutions introduced in *Des*(*i*) sequences is equivalent to twice the number of *s_2_* mutations in pairwise comparison of *Des*(*i*) sequences relative to *Anc*, which happens under the condition that all child sequences in set *Des*(*i*) (*i* = 1, 2, ..., 100) are generated independently of each other. We used this property in the calculation of the average number of mutations between *Des*(*i*) sequences.

Programs developed in paragraphs 4.6.2 and 4.7 can be obtained from the site https://github.com/katri2/MAHDS_addition (21 March 2022). 

## Figures and Tables

**Table 1:** Mean *Z* and *CS* values for BAliBASE protein families’ alignments produced by MAHDS with different *K_d_*, *d*, and *e* parameters.

*R_L_*	*K_d_*	*d*	*e*	*Z*	*CS* (Bali_Score)	*CS* (Bali_Score_Reliable)
5.0	−1.0	40.0	5.0	178.02	0.32	0.43
	−1.0	40.0	4.0	180.08	0.33	0.45
	−1.0	40.0	2.0	182.26	0.39	0.49
	−1.0	40.0	1.0	180.95	0.43	0.52
	−1.0	40.0	0.2	146.40	0.44	0.53
	−2.0	40.0	2.0	172.64	0.32	0.41
	−2.0	28.0	2.8	178.77	0.35	0.45
	−2.0	28.0	0.7	126.46	0.44	0.52

**Table 2:** Mean *Z* and *CS* values for BAliBASE protein families’ alignments produced by different methods.

Methods	*Z*	*CS* (Bali_Score)	*CS* (Bali_Score_Reliable)
MAHDS	180.95	0.43	0.52
T-Coffee	115.31	0.81	0.87
MUSCLE	158.60	0.73	0.80
PRANK	65.50	0.64	0.70
Clustal Omega	116.95	0.81	0.85
Kalign	131.51	0.75	0.82
MAFFT	125.32	0.80	0.85

**Table 3:** *Z* values for multiple alignments of 81 *Des* sets built using MAHDS (*Z* values > 10.0 are in bold).

Indel Count	Indel Length	*x* = 0.6	*x* = 1.2	*x* = 1.8	*x* = 2.4	*x* = 3.0	*x* = 3.6	*x* = 4.2	*x* = 4.8	*x* = 5.4
2	1	**604.3**	**514.0**	**290.5**	**206.8**	**132.4**	**87.0**	**24.4**	**13.1**	7.2
2	5	**761.6**	**476.0**	**302.1**	**208.2**	**113.6**	**85.6**	**21.3**	**11.6**	3.4
2	20	**570.9**	**382.6**	**277.6**	**193.4**	**119.7**	**38.3**	**17.7**	**11.2**	8.1
5	1	**565.0**	**387.4**	**288.3**	**179.1**	**133.0**	**36.1**	**19.2**	**12.7**	8.4
5	5	**570.9**	**366.6**	**266.1**	**174.4**	**114.2**	**27.0**	**14.6**	8.7	6.8
5	20	**504.3**	**355.3**	**218.3**	**141.0**	**57.2**	**22.0**	**12.2**	8.8	6.4
10	1	**596.6**	**373.1**	**232.0**	**145.2**	**82.6**	**21.4**	**13.7**	9.7	6.4
10	5	**557.2**	**342.4**	**230.1**	**129.7**	**33.8**	**16.8**	**11.0**	7.6	6.0
10	20	**353.3**	**233.8**	**144.0**	**39.1**	**22.3**	**13.4**	8.6	7.0	5.2

**Table 4:** *Z* values calculated for multiple alignments of 81 *Des* sets constructed using Clustal Omega and Kalign (*Z* values > 10.0 are in bold).

Indel Number	Indel Length	Clustal Omega			Kalign	
		*x* = 0.6	*x* = 1.2	*x* = 1.8	*x* = 0.6	*x* = 1.2
2	1	**619.8**	**429.2**	**142.5**	**613.9**	**364.9**
2	5	**576.6**	**410.7**	**100.5**	**622.3**	**371.0**
2	20	**520.8**	**331.4**	**45.4**	**559.0**	**284.2**
5	1	**464.4**	**353.3**	−46.8	**603.4**	**300.1**
5	5	**542.5**	**247.3**	−69.7	**571.0**	**207.1**
5	20	**354.5**	**27.1**	−460.1	**381.1**	**16.2**
10	1	**440.2**	**225.8**	−223.1	**477.6**	**148.4**
10	5	**159.0**	**85.8**	−480.6	**415.3**	−73.3
10	20	**130.3**	−209.9	−493.2	**11.5**	−489.0

**Table 5:** *Z* values calculated for multiple alignments of 81 *Des* sets constructed using MAFFT and MUSCLE (*Z* values > 10.0 are in bold).

Indel Count	Indel Length	MAFFT		MUSCLE			
		*x* = 0.6	*x* = 1.2	*x* = 0.6	*x* = 1.2	*x* = 1.8	*x* = 2.4
2	1	**605.3**	**304.4**	**652.9**	**452.1**	**230.0**	**171.3**
2	5	**639.2**	**264.8**	**602.6**	**460.4**	**244.7**	**186.3**
2	20	**582.9**	**231.5**	**568.6**	**408.0**	**177.2**	**97.1**
5	1	**584.0**	**200.1**	**587.0**	**376.9**	**180.0**	**115.4**
5	5	**482.2**	**111.8**	**523.6**	**331.0**	**168.8**	**43.4**
5	20	**357.0**	−67.5	**456.1**	**193.3**	**24.6**	−73.1
10	1	**433.7**	**62.3**	**485.6**	**216.2**	**108.7**	**60.3**
10	5	**344.7**	−52.5	**472.4**	**201.3**	**93.4**	−65.5
10	20	**71.5**	−406.8	**223.0**	**44.8**	−77.9	−90.5

**Table 6:** *Z* values calculated for multiple alignments of 81 *Des* sets built using PRANK and T-Coffee (*Z* > 10.0 are in bold).

Indel Count	Indel Length	PRANK		T-COFFEE		
		*x* = 0.6	*x* = 1.2	*x* = 0.6	*x* = 1.2	*x* = 1.8
2	1	**658.7**	**388.1**	**601.28**	**492.4**	**298.7**
2	5	**608.8**	**375.9**	**530.01**	**363.3**	**130.6**
2	20	**555.7**	**347.9**	**467.06**	**285.6**	**91.6**
5	1	**576.2**	**284.1**	**607.94**	**401.3**	**271.2**
5	5	**515.6**	**307.3**	**311.63**	**62.5**	−167.5
5	20	**468.2**	**164.0**	**168.84**	−78.8	−355.1
10	1	**462.5**	**125.5**	**479.07**	**314.6**	**147.1**
10	5	**412.8**	**113.4**	−18.4	−260.7	−387.6
10	20	**210.0**	−225.7	−133.27	−415.8	−905.0

**Table 7:** Performance of MAHDS, T-Coffee, and MUSCLE in building MSAs of low identity protein families (*Z* values > 10.0 are in bold). The first two families are taken from https://mizuguchilab.org/cgi-bin/homstrad/browse.cgi (accessed on 5 June 2021) The remaining 19 were obtained from https://pfam.xfam.org/ (accessed on 7 June 2021). The protein family names obtained from the Pfam database are shown in Table 8.

Name/ Accession Number	Number of Sequences	Average Length	Average % Identity	MAHDS			T-Coffee			MUSCLE		
				*Z*	Gap Openings	Gaps	*Z*	Gap Openings	Gaps	*Z*	Gap Openings	Gaps
Fibronectin type 3 domain	13	122	17%	−7.3	63	1465	−12.5	325	1004	−10.9	154	598
PH domain	14	98.0	16%	−12.9	71	2035	−17.6	221	2011	−7.7	172	1387
PF00915	44	234.9	17%	**55.5**	2962	182,664	−88.4	7841	802,423	−15.9	2775	124,031
PF02950	9	76.0	14%	−6.93	33	1061	−13.4	103	296	−2.9	45	188
PF06653	210	162.2	18%	**75.9**	5666	393,226	−124.7	13,698	648,158	**16.4**	6557	380,093
PF07611	97	300.1	18%	**119.2**	5651	163,051	−11.7	10,943	197,366	**88.33**	3743	97,553
PF07622	97	273.6	19%	**114.8**	6896	343,751	−33.5	15,437	616,470	**105.2**	5865	273,614
PF08928	182	120.9	18%	**151.8**	9934	235,705	**28.0**	11,885	374,657	**135.0**	7699	210,857
PF09624	101	144.6	17%	**69.9**	2198	43,877	−0.2	3278	58,118	**74.4**	1459	27,515
PF09987	22	223.7	14%	**15.9**	192	14,967	−14.8	747	8978	**27.2**	290	4748
PF10734	219	80.5	19%	**49.4**	2284	172,735	−77.6	8520	390,912	**24.5**	4601	301,122
PF10805	181	96.9	16%	**69.6**	1602	67,600	−34.7	6415	147,729	**64.7**	3807	75,691
PF10846	285	226.6	12%	**61.1**	13,166	607,607	−91.6	55,603	>5 × 10^6^	−6.5	14,604	778,013
PF10895	33	184.2	17%	**18.7**	585	17,420	−52.7	1487	29,766	3.8	712	12,837
PF11368	178	228.4	17%	**116.2**	2862	33,214	−91.6	15,145	80,954	**106.8**	4108	29,868
PF13944	185	124.2	18%	**18.0**	9220	381,058	−132.5	18,133	>1 × 10^6^	−22.5	5512	268,531
PF16506	28	282.4	14%	−2.6	265	20,329	−79.6	3052	51,549	−3.5	1224	13,805
PF18406	166	87.3	19%	**86.6**	4073	142,973	−45.1	12,397	460,552	**43.2**	5286	160,922
PF18709	91	257.8	16%	**143.8**	3724	101,986	**15.2**	8700	232,070	**143.5**	3280	97,117
PF19443	216	216.7	17%	**71.4**	22,203	533,793	−56.5	39,783	>1 × 10^6^	**100.5**	15,753	349,836
PF19975	121	229.4	19%	**115.9**	6716	179,105	−96.6	12,219	503,784	**32.5**	5233	205,882

**Table 8:** Protein family names from the Pfam database whose accession number is used in Table 7 are shown in this table.

Accession Number	Name
PF00915	Calicivirus coat proteins
PF02950	Conotoxins
PF06653	Tight junction proteins
PF07611, PF07622	Proteins of unknown function
PF08928, PF09624	
PF10734, PF10805	
PF10846, PF10895	
PF11368	
PF09987	Uncharacterized protein conserved in archaea
PF13944	Calycin-like beta-barrel domain
PF16506	Putative virion glycoprotein of insect viruses
PF18406	Ferredoxin-like domain in Api92-like protein
PF18709	Dynamin-like helical domain
PF19443	DAHL domain
PF19975	Double-GTPase 1
